# The balancing act of dementia care: an ethnographic study of everyday life and relatives’ experiences in a Danish nursing home for people living with advanced dementia

**DOI:** 10.1080/17482631.2020.1815484

**Published:** 2020-09-13

**Authors:** Cathrine Hjorth Malta-Müller, Marit Kirkevold, Bente Martinsen

**Affiliations:** a Department of Public Health, Research Unit of Nursing and Health Care, Aarhus University, Copenhagen, Denmark; b Head of Institute of Nursing and Health Promotion, OsloMet University, Oslo, Norway

**Keywords:** Dementia, Alzheimer’s disease, nursing home, long-term care, everyday life, ethnographic research

## Abstract

**Purpose:**

The purpose of this study was to explore how everyday life was organized in a Danish nursing home for people living with advanced dementia and how relatives experienced their family members’ everyday lives.

**Methods:**

Field notes from participant observations (approximately 160 hours) and transcripts from ethnographic interviews with relatives (9) were analysed thematically in accordance with ethnographic principles.

**Results:**

The analysis revealed one main theme, ‘Enabling a meaningful everyday life in the nursing home’ with two corresponding sub-themes: (1) Structures of daily life: Balancing collective and individual activities and (2) Physical togetherness: Balancing being together and being alone.

**Conclusions:**

The findings showed that everyday life in the nursing home was organized to support a meaningful life for the residents by providing activities and togetherness on a daily basis. While relatives generally appreciated the everyday life experienced in the nursing home, challenges were encountered in connection with the provision of an appropriate balance between levels and types of activities and togetherness for all residents.

## Introduction

Due to demographic changes with ageing populations, the number of people living with dementia is increasing worldwide (Prince et al., 2015). Since most types of dementia have a progressive course, the majority of affected people will at some point require support to maintain an everyday life (Paulsen, 2011). At present, much of this support is provided in nursing homes (Ministry of Health & Age, 2016; Prince et al., 2015).

It is well-known that the cognitive decline caused by dementia makes it difficult to maintain social interactions and initiate meaningful activities (Paulsen, 2011). Consequently, residents with dementia require support to prevent the boredom and inactivity that has been associated with nursing home life (Mjorud et al., 2017; Wood et al., 2009). Thus, people with dementia have previously portrayed everyday life in nursing homes as boring (Mjorud et al., 2017) and as a life of uncertainty, isolation and loneliness in which coping relies on acceptance and attempts to make the best of things (Clare et al., 2008). Particularly, lack of social contact and everyday activities has been described in nursing homes for people with dementia (Cahill & Diaz-Ponce, 2011; Orrell et al., 2008), although these factors are known as important elements of residents’ quality of life (Edvardsson et al., 2014; Moyle et al., 2015). Schreiner et al. (2005) found that nursing home residents with dementia experience more positive affect when they participate in structured recreational activities than when they are unoccupied. However, a Swedish study suggests that the prevalence of residents with dementia partaking in everyday activities on a daily or weekly basis is still low (Edvardsson et al., 2014).

Today, everyday life in nursing homes has gained an increased political and scientific interest in Denmark as well as in other parts of the world. This development has been facilitated by demographic estimates (Prince et al., 2015), descriptions of poor nursing home conditions in the media (Madsen, 2019), and by the growing acknowledgement of person-centred care as best practice (Edvardsson et al., 2008; Manthorpe & Samsi, 2016). Thus, governments around the world are becoming increasingly committed to ensure that dementia care is person-centred, which includes an increased focus on social engagement and meaningful activities (Brooker, 2004, 2007; Edvardsson et al., 2010; Kitwood, 2016).

Meaningful activities can be defined as enjoyable activities that serve to improve either emotional well-being, cognitive status, physical function, or reduce behavioural disturbances (Morley et al., 2014). At present, many types of nursing home activities have been developed and tested in scientific studies. Examples of such activities include cognitive stimulus therapy, physical exercise, reminiscence programmes, animal-assisted therapy, music interventions, sensory gardens, etc. (Gonzalez & Kirkevold, 2014; Leggieri et al., 2019; Morley et al., 2014). Also, engaging in recreational activities and everyday tasks commonly occurring in nursing homes such as laying the table or making coffee has been addressed as meaningful for people living with dementia (Edvardsson et al., 2014). While the positive outcomes related to these types of activities seem promising, studies and descriptions of person-centred care also suggest that nursing home residents with dementia have different perceptions of meaningful activities, wherefore individualized activity-planning is recommended (Edvardsson et al., 2010; Mondaca et al., 2018; Tak et al., 2015). In addition, research shows that residents, relatives and staff may have different views on what constitutes meaningful activities (Harmer & Orrell, 2008; Peoples et al., 2018; Popham & Orrell, 2012). For instance, Harmer and Orrell (2008) found that nursing home residents with dementia experience meaning in activities that address their psychological and social needs whereas staff and relatives view activities that maintain physical abilities as meaningful.

While many previous studies have focused on developing and testing specific types of nursing home activities (Morley et al., 2014) or criticizing institutional routines and lack of individualized activities (Klaassens & Meijering, 2015; Mondaca et al., 2018; Tak et al., 2015), only few studies have explored the conditions of everyday life in well-reputed nursing homes for people living with dementia. In Denmark, a research project recently explored the care in a dementia-specific municipal day care unit and nursing home known for its good reputation (Hansen & Rasmussen, 2018). The findings showed that enabling a good everyday life for users and residents entailed staff creativity and initiation of activities that seemed meaningful in the moment (Hansen & Rasmussen, 2018). In the same way, Peoples et al. (2018) investigated daily life in the first Danish dementia village, established to facilitate a meaningful everyday life for its residents. In this study, the findings indicated that the village concept did not necessarily improve everyday life for people living with advanced dementia, because this group of residents could not use the new facilities on their own.

In the future, more nursing homes are needed to accommodate the growing number of people living with dementia (Prince et al., 2015). Accordingly, acquiring more knowledge about different ways to organize everyday life in these types of institutions is an important step towards future developments. In this study, we aimed at contributing to this development by exploring everyday life in a selected nursing home for people living with advanced dementia in Denmark. This nursing is known for replacing medicine with care and for providing a good and meaningful life for their residents. This public reputation gives rise to increased scientific interest and makes the nursing home an interesting case for in-depth ethnographic exploration. Thus, the purpose of this study was to explore how daily life was organized in this particular nursing home setting, and how relatives experienced their family members’ everyday lives.

## Design and methods

The study had a reflexive ethnographic research design (Hammersley & Atkinson, 2007) and the empirical data material was collected by participant observations and ethnographic interviews with relatives (Hammersley & Atkinson, 2007; Spradley, 2016a, 2016b). This design was chosen to facilitate in-depth exploration of the chosen study setting. Thus, by combining active participation, explorative interviewing and reflexive thinking it was possible to establish new knowledge about everyday life in the context of a nursing home recognized for providing a good and meaningful life for their residents.

### Study setting

The study took place at a small-scale nursing home called ‘Dagmarsminde’ established in 2016 in Denmark. This nursing home is located in the countryside, and has nine rooms available for people (or couples) living with advanced dementia. Daily life is centred around an open-plan kitchen and living room located in the middle of the nursing home. The shared living room is equipped with both old-fashioned and modern furniture. In addition, the nursing home has a ‘well-being room’ with a hot water swimming pool, and a small library in the basement. The outdoor facilities include a wooden terrace and an enclosed garden with animals, a tea house and a swing set. A cat and a dog also live at the facility, and the nursing home has an open-door policy that allows relatives to visit and participate in everyday activities and free meals whenever they want.

Another important contextual information is that the nursing home is privately owned. In Denmark, the majority of nursing homes are owned and run by the municipalities (public). However, more self-governing (non-profit) and private (for-profit) nursing homes are emerging (Hjelmar et al., 2016). A private nursing home has no operational agreement with the local municipality. This arrangement makes it easier for the leadership to implement values and care approaches at their own initiative. Furthermore, private nursing homes are allowed to profit from services, if desirable. People from all parts of the country can apply for residency in a private nursing home if they have been referred to long-term care by their own municipality. Subsequently, residents are selected from the private nursing home’s own waiting list, and care expenses are covered by the resident’s municipality. In addition, each resident pays a monthly rent. Also, many private nursing homes offer extra services for an additional cost. For instance, ‘Dagmarsminde’ provides a “wellness-package” with services like hairdressing, massages, manicure, pedicure and facial treatments.

During the study period, the majority of residents in ‘Dagmarsminde’ had Alzheimer’s disease and severe cognitive impairment. Most residents were women, and many had lived in other care facilities previously. Approximately half of the residents were physically mobile without the need for aids or support when moving into this nursing home. Also, most residents had reduced their use of medications after relocation. Particularly, a decrease in the use of psychotropic medicine and dementia medicine (donepezil, rivastigmine and memantine) was reported by the doctor attached to the nursing home. Staff consisted of registered nurses, social and health care workers, social workers, and staff without formal education. On average, fifteen staff members (including the manager and deputy manager) were permanently employed. Furthermore, a number of substitutes also worked at the facility. Usually, three staff members (including the deputy manager) worked during daytime, three/two during the afternoon/evening and two during the night. However, staffing levels were often supplemented by unsalaried trainees and/or the nursing home leader. Similarly, staffing levels were typically higher in case of complex care situations such as severe behavioural disturbances or end of life care. Also, activities such as trips outside the nursing home facility often entailed more staff. Another notable feature was the high proportion of registered nurses. Thus, an average of four registered nurses (including the manager), equalling approximately 27%, were employed at the same time during the study period.

### Data collection

The empirical data material was collected by CHM-M, and neither of the authors had any relation to the nursing home prior to this research project. Initially, participant observations corresponding to approximately 160 hours were undertaken. Each observation period had an average length of 4.8 hours, and observations took place across all shifts (day, evening & night). However, in accordance with the purpose of the study most observations took place during daytime. To get an overall sense of everyday life in the nursing home observations began as ‘grand tour’ observations (Spradley, 2016b). Gradually, observations became more focused on specific dimensions that appeared to be principal for daily life such as activities, repeated routines and social interactions. In this way, the research process was characterized by an ongoing process of progressive focusing (Hammersley & Atkinson, 2007).

Most observations were structured to follow staff members’ work routines. Thus, the first author had a background education as a registered nurse that gave her access to care situations and allowed her to aim for moderate participation during the observation period (Spradley, 2016b). At the same time, characteristics such as young age and limited experience within the area of dementia care made it possible to pursue a role as novice/student (Hammersley & Atkinson, 2007). However, field roles were continuously negotiated and different levels of involvement were evident throughout the observation period. Particularly, challenges occurred when staff perceived the researcher as an external evaluator. These situations rendered active participation in everyday practices more difficult and further attention had to be paid to the critical process of building trust (Hammersley & Atkinson, 2007). Similarly, attempts to maintain self-conscious awareness and make personal and professional presuppositions explicit was an ongoing challenge. Particularly, discussing ideas and hunches with the co-authors and working with personal memos and analytic notes supported the first author’s reflexivity during this part of the data collection. After each observation period, an expanded account of the concended field notes was written to ensure the highest possible levels of accuracy (Hammersley & Atkinson, 2007; Spradley, 2016b).

In the second part of the data collection process, ethnographic interviews were conducted with nine relatives. For ethical reasons, nursing home residents were not interviewed. Instead, relatives were chosen to represent each resident living at the nursing home facility at this specific point of time. Previous research has shown that relatives who visit regularly tend to possess detailed knowledge about their family members and that they keep an eye on the care when they visit (Davies & Nolan, 2006; Graneheim et al., 2014; Helgesen et al., 2015, 2013). Thus, relatives are in a unique position to catch sight of how daily life affects each resident, if they visit frequently and are engaged in the well-being of their family member. For this reason, all interviewees in this study were adult children, who visited the nursing home on a regular basis. Interviews took place in locations chosen by each relative (relative’s home, relative’s workplace, researcher’s office or meeting room at the nursing home facility) and were based on open explorative questions such as: *“How is your mother/father doing?”* and *“How is everyday life in the nursing home?”* In addition, more direct questions were sometimes used to elaborate on the principal dimensions identified during the participant observations such as: *“I noticed that the same activities are repeated every day. What are your thoughts on that?”* The interviews lasted between 35 and 92 minutes and were audiotaped and transcribed by the first author.

### Data analysis

Working within the frame of reflexive ethnography, analysis is not a distinct stage of the research process (Hammersley & Atkinson, 2007). Therefore, the analysis began through the analytic ideas written down during the participant observations. Afterwards, a more formal analysis inspired by Hammersley and Atkinson (Hammersley & Atkinson, 2007) was undertaken across both field notes and interview transcripts. First, the empirical data material was read and re-read to obtain a thorough understanding of the meaning. Second, a process of developing analytical concepts and categories was initiated during which interesting and stable patterns were identified and highlighted across the data material. This process was repeated until a set of promising categories was developed. Categories were then compared and divided into themes. The same comparative process was repeated several times, resulting in one main theme and two sub-themes. The analysis was primarily conducted by the first author who possessed the necessary contextual understanding (‘head notes’) from the participant observations and interviews to fill in and recontextualise events and utterances (Hammersley & Atkinson, 2007). However, emerging analytic ideas and findings were continuously discussed among all authors until consensus was reached.

### Ethics

All residents and relatives were informed about the research project and provided written or verbal consent. To further safeguard the dignity and integrity of each resident, the researcher payed close attention to potential signs of distress related to her presence and questions during the participant observations. For instance, the researcher sometimes withdrew from specific care situations or kept in the background if her attendance seemed to cause restlessness or other types of challenges. Also, the researcher generally refrained from asking the residents too many questions that could cause confusion or concern. The empirical data material was anonymized prior to publication and all participants had been informed about the publication of the nursing home name. The study was registered at the Danish Data Protection Agency (Journal no.: 2016–051-000001) and reviewed by the National Research Ethics Committee (Journal no.: 18007168).

## Results

In the following sections, the main theme ‘Enabling a meaningful everyday life in the nursing home’ is unfolded in two sub-themes: (1) Structures of daily life: Balancing collective and individual activities and (2) Physical togetherness: Balancing being together and being alone. The two sub-themes illuminate how everyday life in the nursing home was organized and how relatives experienced their family members’ everyday lives.

### Structures of daily life: balancing collective and individual activities

**Figure 1.**
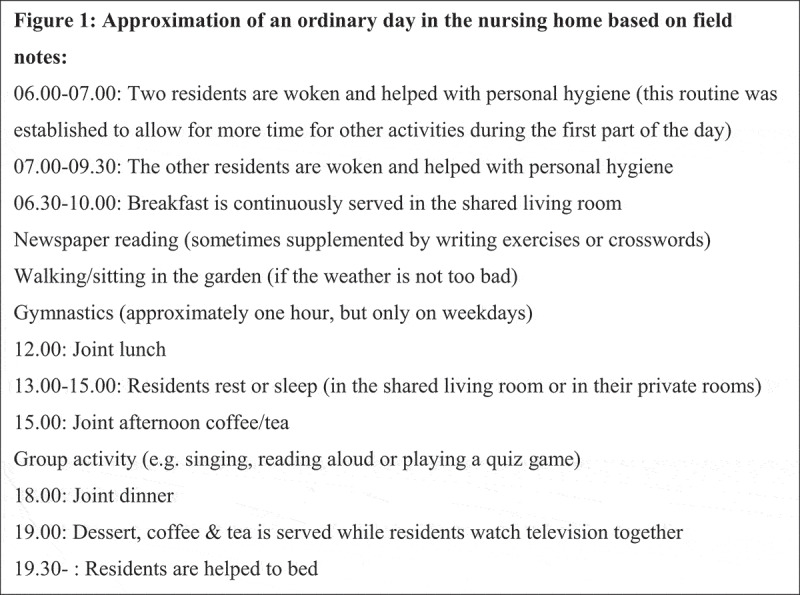
Approximation of an ordinary day in the nursing home based on field notes.

The field notes revealed that everyday life in the nursing home followed the same overall structure organized and maintained by leaders and other staff. First, the residents were woken and helped out of bed within a certain time interval in the morning. Subsequently, they spent the majority of their time in a shared living room located in the middle of the nursing home. Here residents were encouraged to participate in a number of collective activities including meals, newspaper reading and gymnastics. Thus, the same types of activities typically took place at the same time of the day. With few exceptions, residents followed the same rhythm throughout the week (Figure 1).

Overall, relatives experienced that their family members were thriving in the nursing home. This was particularly related to staff members’ facilitation of daily activities. According to relatives, the collective activities ensured that something happened in the residents’ everyday lives and this was perceived as an important feature of the care. To describe the importance of having something to engage in on a daily basis, many relatives referred to previous experiences from other nursing homes. Thus, relatives described how their family members had been offered a minimum of stimulation in their former nursing homes, and that they used to spend much time alone. This had resulted in reactions such as inactivity, boredom, sadness, agitation and/or expressions of wanting to leave the facility. Such experiences served as an argument in favour of the more structured and active everyday life experienced in this nursing home.

Referring to the cognitive decline caused by dementia, relatives explained how their family members were unable to maintain a meaningful everyday life at their own initiative. Thus, having staff to proactively guide and encourage residents to participate in daily activities was experienced as a necessity, as explained by this relative:

Well, the rest of us have a structure as well, right? We use what is called the executive functions, which is the ability to make plans and put them into action. And you lose that [ability] when you have dementia (…), and that’s why I think it’s the best idea in the world to make an external structure (…) It’s part of what people with dementia lose—they lose the structure. Because you need to be able to remember what you have planned to do—and why—and you need to be able to remember what you have done already, and how far you are in the plans. And if you can’t remember those things, then you can’t take any initiative (…), and therefore life becomes nothing. (R4)

Relatives viewed the collective structure as a way of preventing resignation. Thus, management of residents’ lives was perceived as an important and necessary part of proper dementia care. In addition, daily repetition of the same rhythm and the same activities was also viewed as a way of facilitating a sense of security for the individual resident, as described in the following quote:

There’s security in having some routines. Again, it doesn’t need to be new experiences all the time. They [the residents] feel good in the well-known, because they have more than enough to relate to as it is. Just trying to figure out: ‘Why am I here?’ They are constantly running on overtime just trying to figure out: ‘What is going on?’ ‘Where am I?’ They get plenty of stimulants. (R9)

Despite the daily structure, the field notes also revealed some level of flexibility within the regularity. For instance, not all residents participated in the collective activities on a daily basis. Thus, although staff members encouraged participation, they would also accept residents’ declining to participate. In this way, staff tried to achieve a balance between maintaining structure and safeguarding individual needs and wishes. Particularly, relatives experienced that staff were very preoccupied with getting to know their specific family member and utilizing this knowledge to guide their daily work routines. Thus, even though collective activities were a stable and predominant part of everyday life, room was left for individual activities as well. These types of activities included having a private talk, helping out with the animals, laying the table, ironing staff working clothes, arranging flowers, going for a walk, taking a swim in the hot water swimming pool, etc.

Individual activities were initiated by staff for different reasons. On some occasions, residents were asked to help with everyday tasks that staff knew they had formerly enjoyed (e.g., baking or ironing). Similarly, staff sometimes asked a resident to undertake an activity that the person was still good at (e.g., singing or reading). In addition, individual activities were offered simply to promote a resident’s sense of well-being (e.g., swimming/bathing in the hot water swimming pool or getting a manicure) or to reduce behaviours such as restlessness or sadness. In this way, individual activities were used to promote each resident’s capabilities, identity and well-being. Accordingly, relatives viewed these types of activities as important signs of good care, because they allowed their family members to feel as valuable human beings:

… I heard that she [a former gym instructor] introduced some new exercises that they didn’t know (laughing), and that’s … She loves telling how they [the staff] ask her about different things, and it gives her so much self-esteem that she’s needed (…), and she still talks about it, and then she says: ‘I don’t charge anything’ (laughing), and that’s just great. (R9)

Usually, individual activities took place in between or at the same time as the collective activities. Thus, a staff member would typically withdraw from the community with one of the residents. This flexibility was possible because of stable staffing ratios, and shortage of staff was not documented during the study period. Yet, balancing collective and individual activities for all residents at the same time could still be challenging, and sometimes a resident ended up participating in a collective activity even though he or she did not really want to:

[It’s approximately four o’clock in the afternoon. Most of the residents sit together at one of the dining tables in the shared living room. They begin to sing old Danish songs guided by one of the staff members. While they sing, one resident keeps asking a staff member if she can go now? (She needs support to move) The staff member repeatedly asks her, if they can sing one more song first? The resident consents, but after the next song she asks again …] (Field note 20)

Although these types of challenges were not predominant in the field notes, the balancing act of ensuring appropriate activities for all residents at the same time was also referred to by relatives. Thus, some relatives believed that their family members could benefit from taking part in more or other activities than those already offered, and disagreements as to whether trips outside the nursing home facility were good for the residents were also identified in the data material. Thus, some relatives insisted on taking their family members on trips and would also like staff members to arrange more trips outside the nursing home, whereas others emphasized the positive aspects of having their family members stay in the same place and follow the same rhythm every day. Hence, while relatives generally agreed on the important role of activities in the residents’ everyday lives, they did not always agree on the amount and the balance between collective and individual activities:

The way I see it, my mother is one of the most well [residents] in there, so of course she also needs something more. It’s not that the others don’t need something, but maybe she needs to get a little more out for a walk or … That something happens. That she can participate. (…) I know that she irons their working clothes and stuff like that, but maybe she could also fetch eggs and … Well, participate some more maybe. (R2)

In sum, this part of the analysis revealed that daily activities played an important role in the organization of a meaningful everyday life in the nursing home. Particularly, staff members’ attempts to achieve an appropriate balance between collective activities (regularity) and individual activities (flexibility) was important from the relatives’ point of view.

### Physical togetherness: balancing being together and being alone

The structures of daily life entailed that physical togetherness was an unavoidable part of everyday life in the nursing home, and especially the presence of staff was an important reason for residents to reside in the shared living room. According to nursing home policy, at least one staff member should be present in the shared living room at all times. Also, staff did not have any defined breaks, but spent the majority of their working hours together with the residents. These organizational features had practical as well as emotional implications for the residents. Thus, relatives viewed the physical closeness of staff as a way of ensuring the ability to respond to each resident’s needs whenever required. This was confirmed by examples from field notes, where staff often were able to identify and respond to tacit quests such as the need to visit the restroom or take a rest. Similarly, relatives experienced that personalized interactions in the shared living room had a positive impact on their family members’ mental well-being. Thus, staff continuously initiated verbal and nonverbal interactions that corresponded well with each person’s personality and life story. In this way, background knowledge about the individual resident was used to support personal well-being. For instance, having staff around who responded to troublesome questions could have a calming impact on a resident’s state of mind, as explained by this relative:

… [When] someone responds to your questions then you feel at ease, and that’s what we have experienced. In the former nursing home, she kept writing notes like: “Remember … ”, “Please don’t forget me”, “Where am I?” All kinds of notes in all kinds of places, and that has stopped completely. (R9)

Relatives experienced staff members’ attempts to provide personalized interactions in the shared living room as a token of genuine care. Accordingly, field notes uncovered that when staff talked with the residents, called them sweet things like *“honey”* or *“sweetheart”* or physically touched them (stroked their hands or hugged them) the residents appeared to thrive. However, spending time in the shared living room also entailed spending time with other residents. Thus, while relatives generally viewed the staff-resident interactions as a meaningful contribution to everyday life, some ambiguity characterized their considerations regarding the resident-resident interactions. Although different strategies such as predetermined sitting arrangements were applied to support positive resident-resident interactions, relatives viewed these interactions as both positive and negative. Some relatives described how their family members had regained a social life and developed new friendships in the nursing home. In this way, sharing a daily life with other residents could positively influence a resident’s well-being, as described by this relative:

My mom used to be very social. She loved to teach gymnastics, had many friends, and was very outgoing. But for many years she didn’t see anyone. However, because of the community in this nursing home she regained her social life, and we were able to recognise some of her old characteristics. She was thriving again. (R9)

On the other hand, resident-resident interactions could also escalate into irritation and agitation. For instance, one relative described how her father got really annoyed with another resident during lunch because the resident kept asking the same questions over and over again. Likewise, a relative described how her mother got frustrated when other residents intruded on her private relations:

When we visit, she prefers to have us on her own. Because [when we sit in the living room] her “friend” comes over and wants to say hallo as well, and then it’s quite obvious that my mom is doing like this: [The relative is turning her shoulder away]. (R5)

Particularly, the physical presence of staff played an important role for the dynamics within the group of residents, and staff were often able to mediate resident-resident interactions and facilitate a calm and positive atmosphere in the shared living room. Thus, field notes showed that most interactions in the nursing home somehow involved a staff member. Sometimes residents talked with one another for shorter periods of time, but much of the time the conversations were guided by a staff member. For instance, residents often sat in silence at the dining tables until a staff member sat down and facilitated a conversation. The importance of staff facilitation was further underlined by the restlessness that easily arose if all staff members by mistake left the shared living room at the same time:

[It’s afternoon. I’m sitting at one of the dining tables in the shared living room together with most of the residents. At some point, I find myself alone in the living room together with the residents and a young relative. After a short moment one of the residents gets up from her chair. She begins to walk around and the relative whispers to me: ‘Do you think you can help her?’ I walk over and ask the resident if she wants to sit down again? Or maybe go into her room? I can’t really understand what she says and she seems to get more and more irritated with me …] (Field note 13)

In spite of staff members’ ongoing attempts to facilitate a calm and positive atmosphere, residents sometimes refused to stay in the shared living room. For instance, one relative described how her mother did not always want to drink coffee with the other residents and listen to all their *“rubbish”*. The same experience was articulated by another relative, who described how his mother would sometimes refer to the other residents as *“crazy people”* and leave the shared living room to spend some time on her own:

She has a good relation to some of them [the other residents], and then sometimes she says: ‘It’s a bunch of crazy people living here’ (…) Anyway, she likes some of them, and then sometimes, I think, she just needs to withdraw and be alone (…) Then she’ll go into her room, or into the garden to check on the animals if it’s not too cold, or just sit in a chair … (R7)

Withdrawal manifested itself in different ways. Some relatives described how their family members benefitted from leaving the shared living room and spending some time alone (e.g., taking a nap in one’s private room), but most relatives emphasized that their family members did not thrive in their own company for longer periods of time. Accordingly, the field notes showed that most residents tended to return to the shared living room after shorter periods of time. Instead, relatives underlined the importance of being able to be alone while being together. This was referred to as *“staying on the sideline”* and entailed periods during the day where staff encouraged and guided the individual resident to sit in a chair, take a nap in the sun lounge, or in other ways be present without directly engaging in interactions or activities with other residents or staff members. According to relatives, this allowed the individual resident to feel as an integrated part of everyday life—still experiencing stimulation and a sense of belonging—without the pressure of having to contribute. This was for instance, reflected on in the following quote:

… the size of the living room also makes it possible to just be there on the sideline. I can see that’s what my father does a lot (…), and then he is still able to be a part of what’s going on. Or the fact that some of them rest in the sun lounge and stuff like that. (…) Just sitting and being part of a community on the sideline, where there are no demands or things like that—that must be lovely as well, right? (…) Because I have seen the alternative. That is sitting in a room without any external stimulation, and then just sit there, with no ability to structure your life or take initiative or … We create our own stimulating situations where we get stimulated in one way or the other, but you can’t do that anymore when you’re in that situation. (R4)

In sum, this part of the analysis revealed that physical togetherness was another principal feature of the organization of a meaningful everyday life in the nursing home. While relatives generally experienced positive outcomes related to the staff-resident interactions in the shared living room, the analysis also uncovered that residents were not always perceived to thrive in the intense company of each other. Particularly, staff members’ role as facilitators and the possibility of *“staying on the sideline”* were important from the relatives’ point of view.

## Discussion

The findings showed that daily life in the nursing home was characterized by two principal features, which were used by leaders and other staff to enable a meaningful everyday life for residents.

The first principal feature revolved around a stable collective structure applied to provide activities for all residents on a daily basis. Interestingly, relatives perceived the routine of this structure as a positive part of everyday life in the nursing home. This contrasts with previous studies, where routinization of everyday life in nursing homes has been criticized, because residents are deprived autonomy to make choices on their own (Boelsma, Baur, Woelders & Abma, 2014; Cooney, 2012; Harnett, 2010; Persson & Wästerfors, 2009). The same criticism stems from recent studies on dementia care (Klaassens & Meijering, 2015; Mondaca et al., 2018; Nakrem et al., 2013; Tak et al., 2015). However, this discussion is far from new. Already in the 1960s, the seminal work of Erving Goffman (1961) and his theory of ‘total institutions’ initiated a debate about routinization in institutions. Based on his ethnographic studies of persons with mental diseases and other inmates, Goffman argued that people in ‘total institutions’ experienced depersonalization since everybody were treated similarly and were required to do the same things at the same time and place (Goffman, 1961). Although Goffmann did not explicitly discuss nursing homes as ‘total institutions’, findings from other ethnographic studies in nursing homes much resembled his findings (Clark & Bowling, 1990; Gubrium, 1975; Öhlander, 1996; Townsend, 1962). While we acknowledge the negative consequences associated with extreme routinization, our findings suggest that people living with advanced dementia in a nursing home context may benefit from some level of collective structure. Thus, relatives participating in this study emphasized that their family members were not able to make choices on their own any longer and that their lives were likely to turn into nothing if staff did not maintain daily routines and collective activities. In this way, our findings add an interesting perspective to the ongoing discussion by highlighting the more positive aspects associated with routinization. According to social scientists, everyday life can be described as the life people live every day, and the activities they engage in. These activities are typically taken for granted, but at the same time they enable us to maintain a fundamental sense of predictability and stability in life (Bech-Jørgensen, 1994; Scott, 2009). While dementia interferes with the personal ability to maintain one’s former everyday life, findings from this study indicate that a sense of meaning and security can be maintained when staff safeguard routines and activities in residents’ lives.

On the other hand, our study also revealed that providing meaningful activities was not just a matter of collective routines. Instead, an important finding was how individualism was recognized and practiced within the daily routines. Particularly, staff in the nursing home tried to maintain a balance between collective and individual activities. For instance, the findings showed that staff made room for individual activities by involving residents in commonly occurring events such as ironing, baking or laying the table. According to relatives, these activities could support a sense of well-being for each resident. This adds to previous research, where people with dementia have emphasized the importance of still being able to contribute (Moyle et al., 2011). Likewise, Edvardsson et al. found that partaking in household activities was associated with improved quality of life in people living with dementia in residential aged care facilities in Sweden (Edvardsson et al., 2014). However, our findings also uncovered that the balance between collective and individual activities sometimes was challenging to maintain for all residents at the same time, and some relatives believed that their family members could benefit from participating in more individualized activities. The importance of individualized activities has been established in previous studies (Mondaca et al., 2018; Tak et al., 2015) and in descriptions of person-centred dementia care (Edvardsson et al., 2010). However, since all nursing homes by definition are institutions responsible for the well-being of multiple people with multiple needs, it may seem impossible to fulfil the specific wishes and preferences of each individual. In that respect, our findings suggest that individualized care can also be part of a collective everyday life. Thus, while staff in the nursing home worked to maintain a collective structure, they also utilized their knowledge about the individual residents to initiate one-to-one interactions in the shared living room. According to relatives, these interactions supported each resident’s identity and well-being. This is in line with previous results, where the use of biographical information has been described as an effective way of initiating meaningful conversations as part of everyday care routines, and thereby improve the overall well-being of residents with dementia (Brown Wilson et al., 2013). Also, individualized ‘small talk’ in a shared living room may be considered an important contribution towards the achievement person-centred dementia care (Edvardsson et al., 2010).

The second principal feature that characterized everyday life in the nursing home was closely related to the first, and has to do with the high levels of physical togetherness observed during participant observations. Thus, since everyday routines primarily revolved around activities in the shared living room, being physical together was an unavoidable part of residents’ lives and the findings indicated that this type of organization relied heavily on staff members’ role as facilitators. Thus, a special feature of the nursing home was that at least one staff member should be present in the shared living room at all times. According to relatives, this feature promoted personalized interactions between residents and staff, and supported a calm and positive atmosphere. This corresponds with findings from a secured nursing home ward in the Netherlands, where the presence of staff also had a positive effect on the atmosphere in shared spaces (Klaassens & Meijering, 2015). Also, previous studies have indicated that staff play an important role as facilitators of social connectedness in long-term care (Buckley & McCarthy, 2009) and that nursing home residents prefer the company of staff to that of fellow residents (Buckley & McCarthy, 2009; Hauge, 2004; Hauge & Heggen, 2008). For instance, an ethnographic study in a Norwegian nursing home with a mixed resident population revealed that residents preferred social interactions with staff and that physical mobile residents ‘ran away’ from the shared living room whenever staff left (Hauge, 2004; Hauge & Heggen, 2008). That social interactions between residents can be fragile and may suffer from communicative collapse (Hauge & Heggen, 2008) was also seen in our study. Thus, residents in the nursing home would sometimes get irritated with each other and withdraw from the shared living room. Likewise, not much interaction occurred between the residents without the active participation of staff. In this way, our findings further underline the important role staff play in dementia care.

Even though being together in the shared living room entailed some challenges, this study also found that residents were not perceived to thrive in their own company for longer periods of time, and that physical mobile residents tended to return to the shared living room. This corresponds with findings indicating that people living with dementia in nursing homes long for social contact (Cahill & Diaz-Ponce, 2011; Moyle et al., 2011). On the other hand, it is also well-known that too much (or too little) external stimulation can contribute to ‘Behavioural and Psychological Symptoms of Dementia (Cerejeira et al., 2012). In this regard, the present study demonstrates an interesting intermediate position. Thus, our findings illustrate that allowing residents to be alone and withdraw from direct social interactions while still being in the same room can support an appropriate balance between under- and overstimulation for people living with advanced dementia.

### Methodological considerations

In this study, the combination of participant observations and ethnographic interviews with relatives proved to be a valuable approach that allowed a deeper understanding of everyday life in the context of a nursing home for people living with advanced dementia. Also, the first author’s background as a registered nurse was considered a strength that supported her assessments of potential signs of distress related to her presence and questions during the participant observations. At the same time, we recognize that the risk of taking things for granted is far more imminent when studying one’s own culture (Wadel & Fuglestad, 2016).

Since people with advanced dementia are particular vulnerable participants in research, formal interviews were not conducted with any residents in this study. However, safe and inclusive research practices for interviewing people with dementia are emerging (Drageset, 2019; Novek & Wilkinson, 2019). Thus, a suggestion for future research may be to further promote the voices of the affected people by including use of formal interviews. Finally, we acknowledge that this study represents practices and experiences from a selected nursing home. However, this does not mean that the relevance is local. Today, dementia care is widely debated across the world. To qualify this debate new perspectives and pertinent questions are continuously needed. By contributing with knowledge about everyday life from one nursing home this study offers points of contrast, comparison and reference for current discussions and future research.

## Conclusion

The findings from this study showed that activities and togetherness were used by leaders and other staff in an effort to enable a meaningful everyday life for residents living with advanced dementia in the selected nursing home. Overall, relatives believed that this organization contributed to a good life for their family members. However, both features entailed a continuum, and staff became important facilitators of a suitable balance between the outer poles. This balance could sometimes be challenging to maintain for all residents at the same time.
